# *Fasciola spp*: Mapping of the MF6 epitope and antigenic analysis of the MF6p/HDM family of heme-binding proteins

**DOI:** 10.1371/journal.pone.0188520

**Published:** 2017-11-21

**Authors:** Victoria Martínez-Sernández, María J. Perteguer, Mercedes Mezo, Marta González-Warleta, Teresa Gárate, M. Adela Valero, Florencio M. Ubeira

**Affiliations:** 1 Laboratorio de Parasitología, Facultad de Farmacia, Universidad de Santiago de Compostela, Santiago de Compostela, Spain; 2 Centro Nacional de Microbiología, Instituto de Salud Carlos III, Majadahonda, Madrid, Spain; 3 Laboratorio de Parasitología, Centro de Investigaciones Agrarias de Mabegondo, INGACAL, Abegondo (A Coruña), Spain; 4 Departamento de Parasitología, Facultad de Farmacia, Universidad de Valencia, Burjassot, Valencia, Spain; California Northstate University College of Medicine, UNITED STATES

## Abstract

MF6p/FhHDM-1 is a small cationic heme-binding protein which is recognized by the monoclonal antibody (mAb) MF6, and abundantly present in parenchymal cells and secreted antigens of *Fasciola hepatica*. Orthologs of this protein (MF6p/HDMs) also exist in other causal agents of important foodborne trematodiasis, such as *Clonorchis sinensis*, *Opisthorchis viverrini* and *Paragonimus westermani*. Considering that MF6p/FhHDM-1 is relevant for heme homeostasis in *Fasciola* and was reported to have immunomodulatory properties, this protein is expected to be a useful target for vaccination. Thus, in this study we mapped the epitope recognized by mAb MF6 and evaluated its antigenicity in sheep. The sequence of the MF6p/FhHDM-1 ortholog from *F*. *gigantica* (MF6p/FgHDM-1) was also reported. By means of ELISA inhibitions with overlapping synthetic peptides, we determined that the epitope recognized by mAb MF6 is located within the C-terminal moiety of MF6p/FhHDM-1, which is the most conserved region of MF6p/HDMs. By immunoblotting analysis of parasite extracts and ELISA inhibitions with synthetic peptides we also determined that mAb MF6 reacted with the same intensity with *F*. *hepatica* and *F*. *gigantica*, and in decreasing order of intensity with *C*. *sinensis*, *O*.*viverrini* and *P*. *westermani* orthologs. On the contrary, mAb MF6 showed no reactivity against *Dicrocoelium dendriticum* and *Schistosoma mansoni*. The study of the recognition of peptides covering different regions of MF6p/FhHDM-1 by sera from immunized sheep revealed that the C-terminal moiety is the most antigenic, thus being of potential interest for vaccination. We also demonstrated that the production of antibodies to MF6p/FhHDM-1 in sheep infected by *F*. *hepatica* occurs relatively early and follows the same pattern as those produced against L-cathepsins.

## Introduction

Fascioliasis (= fasciolosis) is an important emerging food-borne disease caused by the trematode species *Fasciola hepatica* and *F*. *gigantica* [1, 2]. The disease caused by these parasites is important in terms of pathology [3, 4], but also due to the important economic losses it causes on livestock farms worldwide [5–8]. Humans and animals can become infected by ingestion of metacercariae present in wild or cultured freshwater vegetables, although infection by ingestion of contaminated water is also possible [9]. When the metacercariae are ingested by the corresponding definitive hosts, the parasites excyst, cross the wall of the digestive tract and migrate to the liver where they grow continuously until reaching the adult stage in the bile ducts. The adults start egg production within about 8–12 weeks in small ruminants [10, 11] and within 3–4 months in humans [3, 12]. During their migration through the peritoneum and hepatic parenchyma, the young flukes feed and live in an aerobic environment, in contrast to adult parasites, which live in the almost anaerobic environment of the biliary ducts [13–15]. This lifestyle has profound implications for the metabolism of the flukes, as different set of genes must be activated in these organisms depending on the metabolic requirements throughout the life cycle. This is exemplified by the cysteine proteases of the cathepsin family [16], which are secreted by cecal epithelial cells of the flukes [17, 18] and are necessary for digestion of host tissues [19]. While cathepsins B and cathepsin L3 are predominant in the infective larvae (newly excysted juveniles), production of these enzymes decreases as the parasites grow, and cathepsins L1, L2 and L5 are the most secreted by adult flukes [15, 16, 20]. However, unlike cathepsins, other proteins seem to be necessary throughout the entire life cycle of *Fasciola* [20, 21]. This is the case of the MF6p/FhHDM-1 protein of *F*. *hepatica*, which belongs to the MF6p/HDM family of small (8 kDa, 68 amino acids) cationic heme-binding proteins, also reported in other related trematodes such as *Clonorchis sinensis*, *Opisthorchis viverrini* and *Paragonimus westermani* [22, 23]. MF6p/FhHDM-1 is abundant in the excretory-secretory antigens (ESAs) released by the adult parasites when cultured *in vitro* but unlike L-cathepsins, which are released to the external medium by regurgitation of intestinal waste after digestion, this protein is secreted through the tegument [23]. Moreover, we previously observed that MF6p/FhHDM-1 is present in the *Fasciola* ESAs bound to heme, but that the presence of heme does not interfere with the purification of this protein using the IgG1/k mouse monoclonal antibody (mAb) MF6. In addition, we recently demonstrated that the N- and C-terminal regions of MF6p/FhHDM-1 have different functions, with the former being able to interact with cell membranes and the latter able to interact with hemin and perhaps other as yet unknown molecules [24].

In addition to the heme-binding properties, which are relevant for understanding the homeostasis of heme in trematodes, the MF6p/FhHDM-1 protein has also gained interest due to *in vitro* experiments demonstrating that either the entire MF6p/FhHDM-1 protein or its 37-amino acid C-terminal segment have anti-inflammatory properties on macrophages, which could favor parasite survival [22, 25]. Consequently, it can be expected that blocking such protein by antibodies induced by vaccination may be a useful strategy to diminish the infectivity of the parasite. However, since it is unlikely that a single *Fasciola* antigen will protect ruminants against infection by *Fasciola*, locating immunogenic regions of several antigens which can be combined in one or more chimeric complex antigens may be a reasonable strategy for designing future vaccines against this disease. In this line of research, we mapped the epitope recognized by mAb MF6 and evaluated its antigenicity with respect to the full-length protein and the N- and C-terminal moieties of this molecule. In addition, since the genus *Fasciola* includes two pathogenic species *F*. *hepatica* and *F*. *gigantica*, the sequence of the corresponding MF6p/FhHDM-1 ortholog from *F*. *gigantica* (MF6p/FgHDM-1) was now reported for the first time.

## Materials and methods

### Ethics statement

Experimentally infected or immunized sheep were reared and housed at the Centro de Investigaciones Agrarias de Mabegondo (INGACAL-CIAM), A Coruña (Spain) in strict accordance with Spanish and EU legislation (Law 32/2007, R.D. 53/2013 and Council Directive 2010/63/EU). At the end of the experiments, the animals were sedated with xylazine hydrochloride (Rompun®; Bayer, Mannheim, Germany) and then euthanized with an intravenous injection of a solution containing embutramide, mebezonium iodide and tetracaine hydrochloride (T61®; MSD Animal Health, Salamanca, Spain). All procedures regarding animal handling were approved by the Animal Welfare Committee of INGACAL-CIAM.

### Parasites and antigens

Adult specimens of *Schistosoma mansoni* (21 pairs) were kindly provided by Dr. A. Muro, University of Salamanca, Spain. *Calicophoron daubneyi* (2 specimens), *Dicrocoelium dendriticum* (5 specimens) and *F*. *hepatica* (2 specimens) were obtained from naturally-infected animals, at a local abattoir. *F*. *gigantica* (1 adult specimen) was kindly provided by Dr. Santiago Mas-Coma, University of Valencia, Spain. The parasites were washed 3 times with phosphate-buffered saline (PBS, 15 mM sodium phosphate buffer, 135 mM NaCl, pH 7.4) and homogenized for 3 min in a small volume of PBS containing a protease inhibitor cocktail (SigmaFast Protease Inhibitor Tablets, Sigma-Aldrich, Madrid, Spain) in a motor-driven glass-Teflon homogenizer held in an ice bath. The supernatant extracts were recovered by centrifugation, at 13,000 rpm for 15 min at 4°C, and stored at -80°C until use. The protein concentration of each supernatant was determined with the Micro BCA Protein Assay Kit (Pierce; Thermo Fisher Scientific Barcelona, Spain).

The *Fasciola* ESAs were obtained as previously reported [26]. Briefly, live adult flukes were collected from bile ducts of naturally infected cows and washed, first in sterile saline solution containing antibiotics (penicillin/streptomycin) and glucose (2 g/l) at 38°C and then in RPMI 1640 cell culture medium supplemented with 20 mM HEPES, 0.3 g/l L-glutamine, 2 g/l sodium bicarbonate and antibiotics at 38°C under 5% CO_2_ in air. The flukes were then transferred to 75-cm^2^ tissue culture flasks and maintained in culture medium (3 ml/fluke) at 38°C under 5% CO_2_ in air. The cultures were incubated for 24 h, and the medium containing the secreted antigens was removed and centrifuged at 10,000 *g* for 20 min at 4°C in the presence of protease inhibitors (SigmaFast Protease Inhibitor Tablets, Sigma-Aldrich). The supernatant was then passed through a 0.45-μm pore filter disk, concentrated in an Amicon 8050 ultrafiltration cell (Amicon, Inc., Beverly, MA) equipped with a YM10 membrane (10 kDa molecular weight cutoff), dialyzed against PBS, sterilized by filtration and, finally, stored at -80°C until required. The protein concentration was measured as above. The native MF6p/FhHDM-1 protein (nMF6p/FhHDM-1) isolated from *F*. *hepatica* ESAs was purified by affinity chromatography using the mAb MF6, as previously reported [23].

### Synthetic peptides

**Fig 1:**
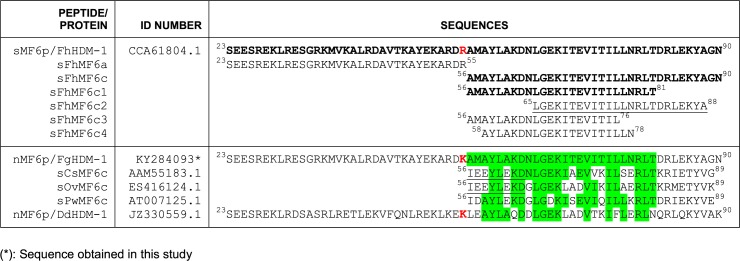
Alignment of *F*. *hepatica* MF6p/FhHDM-1 protein with whole mature sequences and truncated fragments of protein orthologs from other trematodes with sequence homology. Aligned sequences from *F*. *hepatica* (MF6p/FhHDM-1), *F*. *gigantica* (MF6p/FgHDM-1), *D*. *dendriticum* (MF6p/DdHDM-1), *F*. *hepatica* truncated peptides (sFhMF6a, sFhMF6c, sFhMF6c1-4), and truncated peptides corresponding to the C-terminal region from *C*. *sinensis* (sCsMF6c), *O*. *viverrini* (sOvMF6c) and *P*. *westermani* (sPwMF6c) are shown. Synthetic or native (from whole parasite extracts) peptide/proteins used in the study are indicated by adding, respectively, an “s” or an “n” before the corresponding name. The sequences of MF6p/FhHDM-1 recognized by mAb MF6 are highlighted in bold. Amino acid residues in common with the MF6 epitope (residues 56–81) are shown in green. The unique amino acid difference between the *F*. *hepatica* and *F*. *gigantica* protein is indicated in red color. The truncated fragment corresponding to the heme-binding region of MF6p/FhHDM-1 is underlined.

Synthetic sequences corresponding to the complete mature *F*. *hepatica* MF6p/FhHDM-1 protein (sMF6p/FhHDM-1), the N-terminal (residues ^23^S-^55^R; sFhMF6a) and C-terminal (residues ^56^A-^90^N; sFhMF6c) regions, overlapping peptides of different lengths covering the C-terminal region of the protein (sFhMF6c1, sFhMF6c2, sFhMF6c3 and sFhMF6c4), and synthetic peptides corresponding to the C-terminal sequence of *F*. *hepatica* MF6p/FhHDM-1 protein orthologs present in *C*. *sinensis* (sCsMF6c), *O*. *viverrini* (sOvMF6c) and *P*. *westermani* (sPwMF6c) were all obtained (≥95% pure, determined by mass spectrometry) from GeneCust Europe, Dudelange, Luxembourg (see Fig 1 for detailed sequences).

### Production of mAb MF6 antibody

Hybridoma cells secreting mAb MF6 were obtained as previously described [10, 23]. The secreting hybridoma cells were grown intraperitoneally in Pristane-primed BALB/c mice, and the anti–*F*. *hepatica* IgG1/ĸ antibodies were purified from the ascitic fluid by affinity chromatography on a protein G column (HiTrap Protein G, GE Healthcare, Madrid, Spain) according to the manufacturer’s protocol.

### Extraction of *F*. *gigantica* messenger RNA (mRNA)

An adult specimen of *F*. *gigantica* was obtained and classified as previously described [4, 27], and homogenized to a fine powder in a mortar containing liquid nitrogen. Then, the mRNA was extracted with the FastTrack 2.0 mRNA Isolation kit (Invitrogen Inc, Carlsbad, CA) following the protocol established by the manufacturer.

### Synthesis of *F*. *gigantica* complementary DNA (cDNA)

A cDNA library was prepared from 1.8 μg of *F*. *gigantica* mRNA by using the Marathon cDNA Amplification kit (Clontech Laboratories Inc, Mountain View, CA). AP1 sequence adapters were ligated at the ends of the synthesized cDNAs. All procedures were performed according to the manufacturer’s recommendations.

### Cloning of the *F*. *gigantica* MF6p/FgHDM-1 gene

The MF6p/FgHDM-1 gene was amplified by RACE-PCR using the above cDNAs as template and a set of primers which include the adaptor primer AP1 (5’-CTAATACGACTCACTATAGGGC-3’) and two primers coding for both amino and carboxy ends of the MF6p/FhHDM-1 homologous gene from *F*. *hepatica*. These primer sequences were: forward (7Kd_5_RACE_B), 5’-ATGCGCTTCATTGTTCTTCTCTGTCTTGCTGTGGTCC-3’; and reverse (7Kd_3_RACE_B), 5’- TTAATTTCCCGCGTATTTCTCCAAGCGATCGGTGA-3’. Non-directional cloning of the gene was performed. The amplification product corresponding to the *F*. *gigantica* MF6p/FgHDM-1 gene was ligated directly into the pGEM-T Easy vector by standard protocols [28]. Positive recombinant selection was performed by colony-PCR, via amplification with the universal vector primers. The plasmid DNA was extracted from the cultured recombinants and sequenced according to the method of Sanger [29], with the 3730x1 DNA Analyzer (Applied Biosystems) and following the Big-DyeTerminator Cycle Sequencing Ready Reaction Kit v3.1 (Applied Biosystems, Foster City, CA).

### Experimental and natural infections by *F*. *hepatica*

All sheep were of the Galician autochthonous breed. Serum samples were obtained from 6 experimentally infected 9-month-old lambs raised on a *Fasciola*-free farm (Mabegondo) and subsequently infected once with 100 metacercariae obtained in the laboratory from experimentally infected *Galba truncatula* snails. Serum samples were obtained from each animal just before infection and every 2 weeks for 12 weeks post infection.

Serum samples were also obtained from 15 naturally infected adult sheep (between 3 and 8 years old) raised on a commercial farm in A Pastoriza (Lugo, Spain). The sheep were suffering from chronic fascioliasis, which was confirmed by fecal examination (0.3–9 eggs per gram) and detection of coproantigens by the MM3-COPRO ELISA [10, 30]. The presence of intestinal and lung nematodes was also confirmed in all of them. The samples were stored frozen at -20°C until use.

### Immunization of sheep

Two groups of six lambs (4–6 months old) were immunized in the axillar region with 1 ml of PBS containing 100 μg of the synthetic (1 group) or native (1 group) MF6p/FhHDM-1 protein plus 1 mg of Quil-A (Accurate Chemical & Scientific Corporation, Westbury, NY). Four weeks after the first immunization, the lambs were given a second dose of the same antigens. Finally, two weeks after the second immunization, the animals were bled and the serum samples obtained were stored individually at -30°C for further studies.

### Competitive indirect ELISAs

#### Inhibition of mAb MF6

Polystyrene microtiter plates (Greiner BioOne, Soria-Melguizo, Madrid, Spain) were coated with 100 μl/well of synthetic MF6p/FhHDM-1 at a concentration of 10 μg/ml in TBS buffer (50 mM Tris, 150 mM NaCl, pH 7.3). The plates were incubated overnight at 4°C and were then washed three times with TBS and blocked with 200 μl/well of 1.5% sodium caseinate in PBS for 2 h at room temperature (RT). In parallel, mAb MF6, at a final dilution of 1/200,000, was preincubated with twofold dilutions of each synthetic peptide (sMF6p/FhHDM-1, sFhMF6a, sFhMF6c, sFhMF6c1-4, sCsMF6c, sOvMF6c and sPwMF6c), starting at 20 μg/well in TBS containing 0.05% Tween and 1% dry skimmed milk (TBS-T-SM), for 30 min at RT and shaking at 750 rpm. Aliquots of 100 μl/well of each mixture and non-inhibited mAb MF6 (control) were then added to the ELISA plates. The plates were incubated for 30 min at RT, with shaking at 750 rpm, and were then washed five times with TBS-T and incubated with HRP-labeled goat anti-mouse IgG secondary antibodies (EIA grade, Bio-Rad, Madrid, Spain) diluted 1/3,000 in TBS-T-SM, for 30 min at RT, with shaking at 750 rpm. The plates were washed again and 100 μl of substrate (SigmaFast OPD; Sigma-Aldrich) was added to each well. The reaction was stopped by addition of 25 μl of 3 N H_2_SO_4_ after 20 min at RT, and the optical density (OD) was read at 492 nm. The percentage inhibition was calculated according to the formula [(OD2- OD1)/OD2) x 100], where OD1 and OD2 correspond to the inhibited and non-inhibited mAb MF6, respectively. The mean OD for mAb MF6 without inhibitor (control for maximal signal) was 1.60±0.027.

#### Inhibition of sheep antibodies

ELISA plates were coated with sMF6p/FhHDM-1 as described above. In a preliminary assay, each serum from sheep immunized with nMF6p/FhHDM-1 (n = 6) was tested at dilutions ranging from 1/400 to 1/6,000 in TBS-T-SM in order to obtain an OD near 1. Once selected the optimal dilution, each serum was preincubated individually with the synthetic peptides sMF6p/FhHDM-1, sFhMF6a or sFhMF6c at 5 μM, with both sFhMF6a and sFhMF6c (5 μM each) peptides, or with a large excess of mAb MF6 (dilution 1/1,000), all prepared in TBS-T-SM, for 30 min at RT and shaking at 750 rpm. Then, 100 μl aliquots of each mixture and of non-inhibited sera (controls) were added to each well and the plates were incubated for 30 min at RT, with shaking at 750 rpm. The plates were washed five times with TBS-T and bound sheep IgG antibodies were detected with HRP-conjugated monoclonal anti-sheep/goat IgG (Sigma–Aldrich, 1/30,000 in TBS-T-SM) and OPD. The percentage inhibition was calculated as indicated above.

### Indirect ELISA

The wells of ELISA plates were coated with 100 μl of the synthetic peptides sFhMF6c, sCsMF6c, sOvMF6c or sPwMF6c prepared at a concentration of 10 μg/ml in TBS. The plates were incubated for 2 h at 37°C and were then washed three times with TBS and blocked with 200 μl/well of 1.5% sodium caseinate in PBS for 2 h at RT. Aliquots of 100 μl of sera from sheep immunized with nMF6p/FhHDM-1 (n = 6) or sMF6p/FhHDM-1 (n = 6) and diluted 1/100 in TBS-T-SM were added to the wells of each plate, and were then incubated for 30 min at RT with shaking at 750 rpm. Finally, the plates were washed five times with TBS-T, and bound IgG antibodies were detected as above.

### Capture ELISAs

#### MF6-ELISA

Prior to designing this capture ELISA, we carried out a sandwich ELISA with mAb MF6 (capture) and FITC-labeled mAb MF6 (detection) antibody pairs, to test if there are MF6 epitopes available when the nMF6p/FhHDM-1 present in ESAs is immobilized in the plates by mAb MF6. In this experiment we observed that although the MF6p/FhHDM-1 protein contains a single epitope recognized by mAb MF6 (this study), due to the ability of this protein to oligomerize [22, 24], there are still free MF6 epitopes for other antibodies in capture ELISA. Considering this, the sera from naturally and experimentally infected sheep were analyzed by capture MF6-ELISA, as follows. Polystyrene microtiter plates were coated with mAb MF6 (100 μl/well at 5 μg/ml), incubated overnight at 4°C, washed three times with PBS and blocked with 200 μl/well of 1.5% sodium caseinate in PBS for 2 h at RT. Aliquots of 100 μl of *F*. *hepatica* ESAs at 2.5 μg/ml in PBS containing 0.05% Tween and 1% dry skim milk (PBS-T-SM), or PBS-T-SM only, were added to each well in odd (Ag+) and even (Ag-) plate rows, respectively. The plates were incubated for 30 min at RT with shaking at 750 rpm and were then washed five times with PBS-T, before 100 μl of each sample serum diluted 1/100 in PBS-T-SM was added to each Ag+ and Ag- well. The plates were incubated again and washed as above and specific sheep IgG were detected with HRP-conjugated monoclonal anti-sheep/goat IgG (Sigma–Aldrich) diluted 1/30,000 in PBS-T-SM, and OPD. The OD value for each sample was calculated as OD1-OD2, where OD1 is the value for the Ag+ well, and OD2 is the value for the Ag- well.

#### MM3-ELISA

The same ovine sera tested by MF6-ELISA were analyzed by MM3-ELISA for comparative purposes. This method uses the MM3 monoclonal antibody, which recognizes an epitope present in *F*. *hepatica* and *F*. *gigantica* L-cathepsins, and was performed as the previously reported MM3-SERO ELISA [11] with some modifications. Briefly, polystyrene microtiter plates were coated with 100 μl/well of mAb MM3 (prepared at 5 μg/ml in PBS), incubated overnight at 4°C, washed three times with PBS and blocked with 200 μl/well of 1.5% sodium caseinate in PBS for 2 h at RT. Aliquots of 100 μl of *F*. *hepatica* ESAs at 1 μg/ml in PBS or PBS only were added to each well in odd (Ag+) and even (Ag-) plate rows, respectively. The plates were incubated for 2 h at RT and were then washed three times with PBS, before 100 μl of each sample serum diluted 1/100 in PBS-T-SM was added to each Ag+ and Ag- well. The plates were incubated for 30 min at RT with shaking at 750 rpm, washed five times with PBS-T, and specific sheep IgG was detected as described above. The OD value for each sample was calculated as for the MF6-ELISA.

### Immunoblot analysis

For the immunoblot analysis, 10 μg aliquots of each extract of *F*. *hepatica*, *F*. *gigantica*, *C*. *daubneyi*, *D*. *dentriticum* and *S*. *mansoni* were processed on 10–20% linear gradient polyacrylamide gels, under reducing conditions, following the procedure described by Laemmli [31]. The separated proteins were transferred to PVDF membranes (Immobilon-P, Millipore Ibérica SA, Madrid, Spain) in a Trans-Blot SD transfer cell (Bio-Rad) at 120 mA for 30 min. The membranes were subsequently blocked for 2 h at RT with PBS-T-SM and then incubated with mAb MF6 diluted 1:10,000 in PBS-T-SM for 1 h at RT on an orbital shaker. The membranes were washed three times for 5 min each with PBS-T and then incubated with HRP-labeled goat anti-mouse IgG secondary antibodies diluted 1/1,500 in PBS-T-SM for 1 h at RT. Finally, the membranes were washed again, and the bands were developed using 3,3’-diaminobenzidine tetrahydrochloride tablets (Sigma-Aldrich), following the supplier’s instructions.

### Bioinformatics analysis

The secondary structure of *Fasciola* MF6p/FhHDM-1 and orthologous proteins from other trematodes (*C*. *sinensis*, *O*. *viverrini*, *P*. *westermani* and *D*. *dendriticum*) was predicted using bioinformatics tool JPred 4.0 [32]. *Ab initio* (template-free) 3D protein structure predictions for the above proteins were carried out with the Quark bioinformatics tool [33]. Although this tool has important limitations, as with all *de novo* prediction methods, it is particularly recommended for proteins with ≤100 residues. The PyMOL Molecular Graphics System (Version 1.8 Schrödinger, LLC) was used to visualize the PDB files generated by Quark.

### Statistical analysis

The significance of differences between the antibody response to L-cathepsins and to the nMF6p/FhHDM-1 protein in sheep experimentally infected with *F*. *hepatica*, and of results from ELISA inhibition experiments with sheep sera, was determined using the Tukey-Kramer multiple comparison test. The MM3-ELISA and MF6-ELISA results obtained testing sera from sheep naturally infected with *F*. *hepatica* were compared using the Mann-Whitney test. The statistical analyses were conducted using the GradPad Instat statistical package (GraphPad Software Inc, CA). Differences were considered significant at p <0.05.

## Results

### The epitope recognized by mAb MF6 is located in the C-terminal region of the *F*. *hepatica* MF6p/FhHDM-1 protein

**Fig 2:**
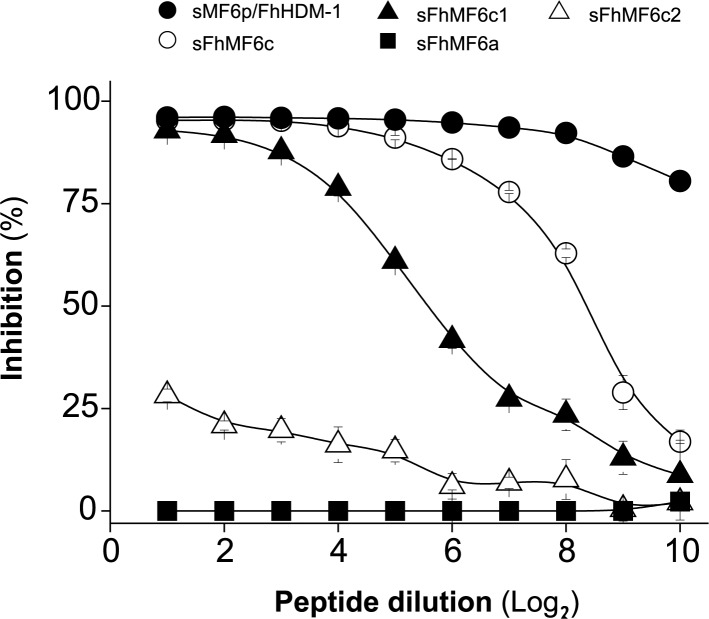
Determination of the region of MF6p/FhHDM-1 recognized by mAb MF6 in competitive ELISA. ELISA plates coated with sMF6p/FhHDM-1 were incubated with mAb MF6 (diluted 1/200,000) previously incubated with twofold dilutions of the synthetic peptides sMF6p/FhHDM-1 (control for maximal inhibition; filled circles), sFhMF6c (residues ^56^A-^90^N; unfilled circles), sFhMF6c1 (residues ^56^A-^81^T; filled triangles), sFhMF6c2 (residues ^65^L-^88^A; unfilled triangles), and sFhMF6a (residues ^23^S-^55^R; filled squares). Peptides sFhMF6c3 and sFhMF6c4 produced the same negative result as sFhMF6a, but for the sake of simplification were not represented here. Data are expressed as percentage inhibition of mAb MF6 by each peptide and are the mean values ± SD of duplicate wells.

To investigate the position of the epitope recognized by mAb MF6 in the MF6p/FhHDM-1 sequence, we designed an inhibition ELISA in which the wells of the ELISA plate coated with sMF6p/FhHDM-1 were incubated with mAb MF6 preincubated with several concentrations of overlapping peptides covering the entire protein sequence (see Fig 1; upper row). Binding of mAb MF6 to the immobilized sMF6p/FhHDM-1 protein was mainly inhibited by sMF6p/FhHDM-1 (positive control for inhibition), but also by peptide sFhMF6c (residues ^56^A-^90^N), which clearly indicates that the epitope is located in the C-terminal region of MF6p/FhHDM-1 (Fig 2). Interestingly, when we evaluated peptides sFhMF6c1 and sFhMF6c2, which are shortened, respectively, by 9 residues from the carboxy or amino region with respect to the sFhMF6c peptide, we observed that the former was still able to effectively inhibit the binding of mAb MF6 to the ELISA plate. Nevertheless, the truncated peptide sFhMF6c2 only produced a 25% inhibition at the maximum concentration. On the contrary, we observed that the N-terminal moiety of MF6p/FhHDM-1 (sFhMF6a) and two truncated peptides (sFhMF6c3 and sFhMF6c4; see sequences in Fig 1) derived from sFhMF6c1 (not shown) were not able to inhibit the binding of mAb to the immobilized sMF6p/FhHDM-1 protein.

### The MF6 epitope is also present in the MF6p/FgHDM-1 protein from *F*. *gigantica* and in orthologous proteins from some related trematodes

**Fig 3:**
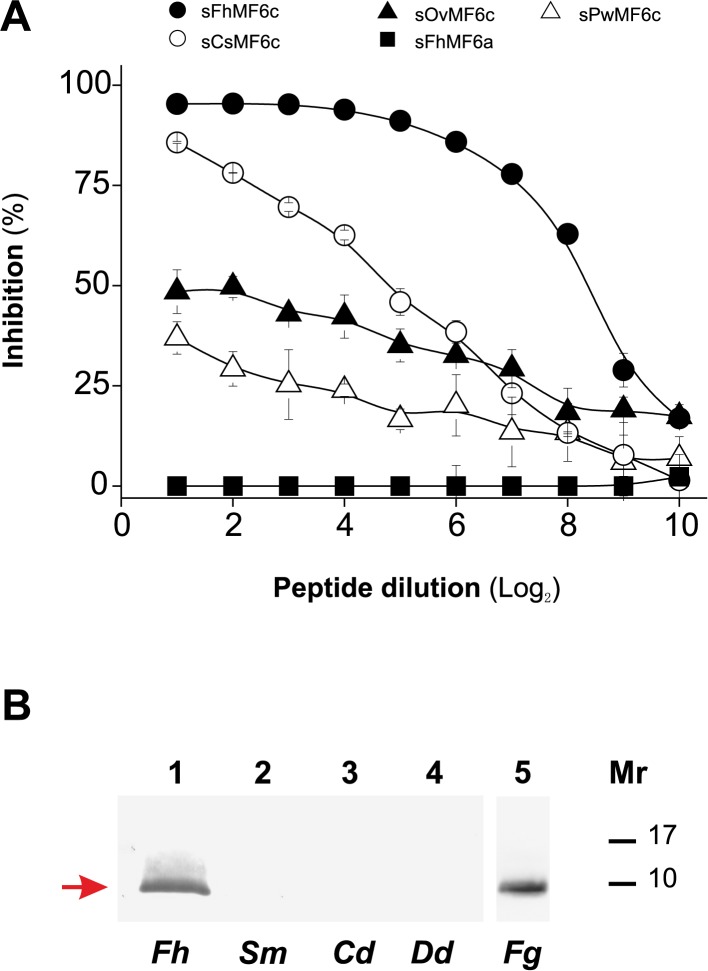
Analysis of the potential cross-reactivity of mAb MF6 with MF6p/FhHDM-1 orthologs from other trematodes. A) Competitive ELISA with synthetic peptides corresponding to the C-terminal region of MF6p/FhHDM-1 orthologs. ELISA plates coated with sMF6p/FhHDM-1 were incubated with mAb MF6 (diluted 1/200,000) previously incubated with twofold dilutions of sFhMF6c (residues ^56^A-^90^N; control for maximal inhibition; filled circles) from *F*. *hepatica*, sCsMF6c (residues ^56^I-^89^G; unfilled circles) from *C*. *sinensis*, sOvMF6c (residues ^56^I-^89^K; filled triangles) from *O*. *viverrini*, sPwMF6c (residues ^56^I-^89^E; unfilled triangles) from *P*. *westermani*, and sFhMF6a (residues ^23^S-^55^R; filled squares) as a negative control. Data are expressed as percentage inhibition of mAb MF6 by each peptide and are the mean values ± SD of duplicate wells. B) Immunoblotting analysis showing the recognition by mAb MF6 of several natural extracts from different trematodes. Lane 1: Fh (*F*. *hepatica*); lane 2: Sm (*S*. *mansoni*); lane 3: Cd (*C*. *daubneyi*); lane 4: Dd (*D*. *dendriticum*); lane 5: Fg (*F*. *gigantica*).

The proteins of the MF6p/HDM family from different trematodes display a high degree of similarity in the C-terminal region (Fig 1). We therefore tested whether synthetic peptides derived from orthologous proteins present in related trematodes also react with mAb MF6 in a competitive ELISA. The results of the inhibition ELISA show that peptides corresponding to the C-terminal region of MF6p/FhHDM-1 orthologs belonging to *C*. *sinensis*, *O*. *viverrini* and *P*. *westermani* are also recognized by mAb MF6 (Fig 3A). However, among them, only the peptide sCsMF6c (from *C*. *sinensis*) was able to inhibit binding of mAb MF6 to the immobilized *Fasciola* sMF6p/FhHDM-1 protein by more than 75%. In addition to the results of the inhibition ELISA, we observed that mAb MF6 was also able to react in Western Blot with whole parasite extracts from *F*. *gigantica* (MF6p/FgHDM-1) but not with those from *S*. *mansoni*, *C*. *daubneyi* or *D*. *dendriticum* (Fig 3B).

Given that the whole extract of *F*. *gigantica* is also recognized by mAb MF6, we used a RACE-PCR to investigate whether the MF6p/FhHDM-1 ortholog in this species (MF6p/FgHDM-1) has the same sequence as *F*. *hepatica*. Comparison of the ORFs of both proteins revealed 5 nucleotide changes in MF6p/FgHDM-1 with respect to MF6p/FhHDM-1, only one of which led to an amino acid substitution (^55^R to ^55^K) in the translated protein sequence. This almost complete sequence identity between both proteins (see Fig 1) explains the identical reactivity observed with mAb MF6 (Fig 3B) and suggests they are key proteins within the genus *Fasciola*. Moreover, the above amino acid substitution of ^55^R by ^55^K is a minor change, as both residues are positively charged and this change was also observed in the sequences from *D*. *dendriticum* and *P*. *westermani* (see Fig 1).

### Key residues of the MF6 epitope are located at the predicted coil and α-helix regions of MF6p/FhHDM-1

**Fig 4:**
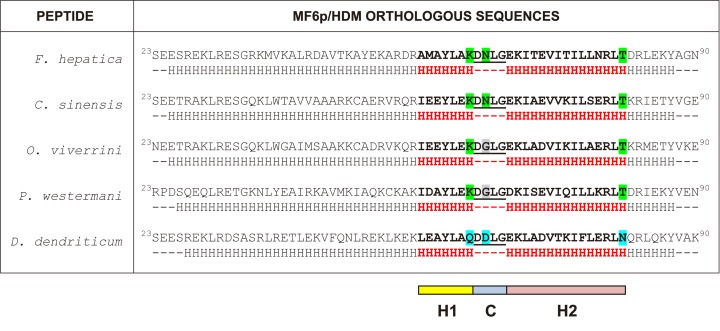
Comparison of the predicted secondary structure of MF6p/FhHDM-1 orthologs from different trematodes. Complete sequences of the mature MF6p/HDM proteins from *F*. *hepatica*, *C*. *sinensis*, *O*. *viverrini*, *P*. *westermani* and *D*. *dendriticum* (residues 23–90). The α-helix (H) and coil regions (C) predicted by J-Pred 4 bioinformatics tool are also represented. The regions H1, C and H2 correspond to the α-helix/coil/α-helix region covering the MF6 epitope. The residue substitutions corresponding to the putative relevant positions ^62^K, ^64^N and ^81^T of MF6p/FhHDM-1 for MF6 binding are shown in different colors.

From a dichotomous view (linear/conformational) the epitope recognized by mAb MF6 would be classified as lineal. However, the relatively large number of residues (n = 26) involved in the binding of mAb MF6 to the MF6p/FhHDM-1 protein and the fact that the inhibitory effect observed in ELISA increases with the length of the peptide sequence (see Fig 2), suggests that the secondary structure of the protein and derived peptides is probably important for MF6 binding. Thus, considering these data, the classification of this epitope as continuous conformational (semiconformational) [34] is probable more correct. In this regard, we used the Jpred 4 bioinformatics tool to compare the secondary structures of MF6p/HDM orthologs from 5 representative trematodes which are well recognized (*F*. *hepatica/gigantica* and to a lesser extent *C*. *sinensis*), weakly recognized (*O*. *viverrini* and *P*. *westermani*) or not recognized (*D*. *dendriticum*) by mAb MF6. The Jpred 4 tool predicted the stretches corresponding to the MF6 epitope (residues ^56^A-^81^T from either MF6p/FhHDM-1 or MF6p/FgHDM-1 proteins; Fig 4, sequence highlighted in bold) as having three differentiated regions. These are a coil (C) region (^63^DNLG^66^) flanked on its left hand (H1) by 7 residues (^56^AMAYLAK^62^) and on its right hand (H2) by 15 residues (^67^EKITEVITILLNRLT^81^), both of which are part of two longer α-helices (Fig 4). The corresponding sequences from the other trematodes (Figs 1 and 4) have several amino acid substitutions in this region with respect to the *F*. *hepatica/gigantica* sequence (8–12 changes), although the size of the predicted coil, as well as the predicted overall secondary structure in this region, was the same as for *F*. *hepatica*.

To obtain more information about which residues are directly involved in the interaction with the paratope of mAb MF6 (hot spot residues) [35], we determined which amino acid changes in the above three regions are associated with a decrease, or a significant loss of inhibitory activity in the competitive ELISA. Since the peptide sFhMF6c2 showed some inhibition, whereas peptides sFhMF6c3 and sFhMF6c4 did not, we concluded that the C-terminal ^79^RLT^81^ sequence of the sFhMF6c1 peptide probably includes an anchor point for mAb MF6. Moreover, within this sequence, ^81^T is probably important since it is present in all peptides from trematodes recognized by mAb MF6, while in *D*. *dendriticum* (not recognized) it was substituted by ^81^N (see Figs 1 and 4). The residue ^64^N present in the coil region (region C in Fig 4) is another anchor point candidate, as it is present in the peptides derived from *F*. *hepatica* and *C*. *sinensis* (good inhibitors), while its substitution by ^64^G (*O*. *viverrini* and *P*. *westermani*) or by ^64^D (*D*. *dendriticum*) leads, respectively, to a significant decrease or complete loss of mAb MF6 recognition (see Figs 3 and 4).

Unlike for the above C and H2 regions of the MF6 epitope, we were not able to clearly identify any anchor residue within the H1 region. In fact, we observed that the H1 region of *C*. *sinensis* (well recognized) and *O*. *viverrini* (poorly recognized) have identical H1 sequences, while the H1 sequence of *D*. *dendriticum* (not recognized) is very similar to that of *F*. *hepatica*. Nevertheless, we could not rule out the possibility that the substitution of ^62^K by ^62^Q in *D*. *dendriticum* (see Figs 1 and 4) may be important for abrogation of MF6 binding.

**Fig 5:**
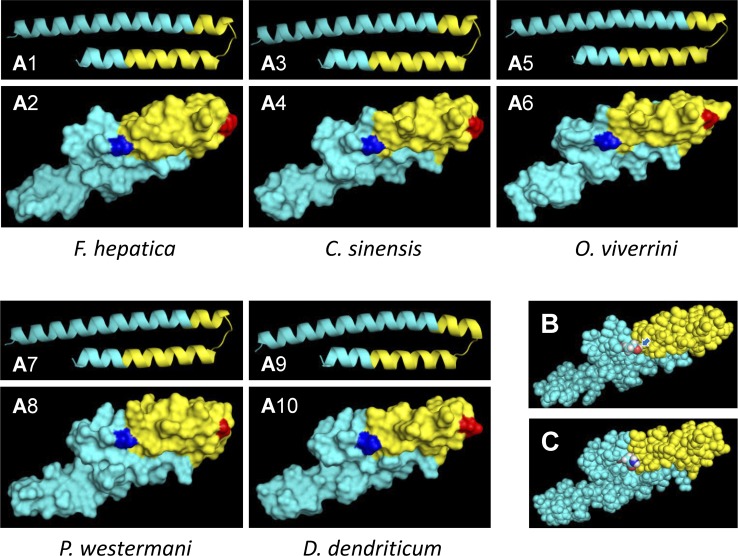
Three-dimensional (3D) structure predictions of MF6p/FhHDM-1 orthologs from different trematodes. A) Predicted PDB structures for MF6p/FhHDM-1 orthologs from *F*. *hepatica*, *C*. *sinensis*, *O*. *viverrini*, *P*. *westermani* and *D*. *dendriticum* using the Quark Online *ab initio* protein folding and protein structure prediction bioinformatics tool. For better visualization, each 3D structure was represented as a cartoon (odd numbers) or as a surface (even numbers). The overall surface structures are shown in cyan, the area corresponding to the MF6-recognized epitope is represented in yellow and the positions corresponding to ^64^N and ^81^T in the sequence of *F*. *hepatica*, assumed to be highly relevant for MF6 epitope conformation, are shown in red and blue, respectively. B) Theoretical 3D structure of the MF6p/FhHDM-1 mature protein (represented as balls) showing the overall structure of the MF6-recognized epitope (shaded in yellow) and the position of the N-terminal ^81^T residue (colored atoms; red = oxygen; white = hydrogen; cyan = carbon). The arrow shows the position of the hydroxyl group of the ^81^T residue. C) Theoretical 3D structure of the MF6p/FhHDM-1 mature protein ortholog from *D*. *dendriticum* (represented as balls) highlighting the equivalent region to the MF6-recognized epitope (shaded in yellow) and the position of the N-terminal ^81^N residue in substitution of ^81^T in *F*. *hepatica* (colored atoms; red = oxygen, see arrow; white = hydrogen; cyan = carbon). All 3D representations of the PDB models obtained with the Quark Online bioinformatics tool were elaborated using the PyMOL Molecular Graphics System.

To facilitate visualization of the region in the *F*. *hepatica* MF6p/FhHDM-1 protein recognized by mAb MF6, we used the *Quark* bioinformatics tool [33] to generate *ab initio* 3D structure predictions for this protein and related homologs. We then used the PyMol bioinformatics tool to represent the corresponding PDB structures. The 3D structures for each protein were represented as a cartoon (panels A1, A3, A5, A7 and A9) and as a surface (panels A2, A4, A6, A8, A10) (Fig 5). In addition to the putative α-helices and the surfaces exposed to the solvent, the area corresponding to the MF6 epitope (shaded in yellow) and the positions of the two putative ^64^N (red) and ^81^T (blue) polar hydrophilic anchors in the C and H2 regions (Fig 4) can also be observed. Finally, in Fig 5C and 5D we presented the predicted surface of MF6p/FhHDM-1 and the corresponding ortholog from *D*. *dendriticum* (represented as balls), respectively, to show the availability of a hydroxyl group in ^81^T (*F*. *hepatica*) which is not present in ^81^N (*D*. *dendriticum*).

### The C-terminal region of MF6p/FhHDM-1 is potentially more antigenic than the N-terminal region of the protein

**Fig 6:**
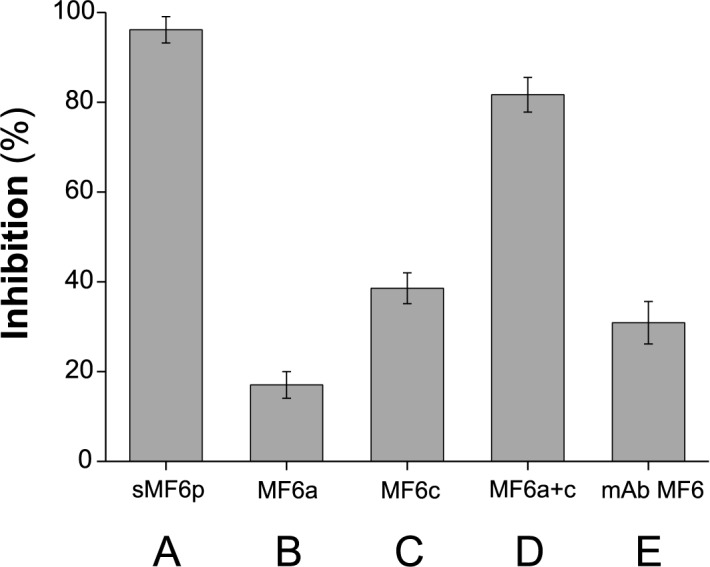
Analysis of the antigenicity of the MF6p/FhHDM-1 protein in sheep. The antibody response from a group of 6 sheep immunized with nMF6p/FhHDM-1 in Quil-A was tested in a competitive ELISA. The experiment was carried out with ELISA plates coated with sMF6p/FhHDM-1 and each serum (diluted at an optimal dilution between 1/400 and 1/6,000) was incubated in duplicate in the absence (control for maximal signal) or in the presence of 5 μM of sMF6p/FhHDM-1 (control for maximal inhibition), sFhMF6a, sFhMF6c, or sFhMF6a plus sFhMF6c. The data shown also include the inhibition achieved with sheep sera in the presence of a large excess of mAb MF6, in order to measure the antigenicity attributable to the MF6 epitope. Data are expressed as percentage inhibition of sheep sera by the protein/peptides or mAb MF6 and are the mean values ± SD of all sheep. The mean OD for the adjusted sera without inhibitor was OD = 0.69 ± 0.07. Abbreviations: sMF6p = sMF6p/FhHDM-1; MF6a = sFhMF6a; MF6c = sFhMF6c; MF6a+c = sFhMF6a plus sFhMF6c. The Tukey-Kramer multiple comparison test revealed statistical differences between all groups (p<0.05 for groups C and D; p<0.01 for the remaining groups).

After defining the region of the MF6p/FhHDM-1 protein covering the MF6-recognized epitope, we investigated its antigenic relevance and that of other parts of the molecule with respect to the entire molecule. For this purpose, we immunized a group of 6 sheep with the nMF6p/FhHDM-1 protein in Quil-A (twice, with an interval of one month) and tested the resulting sera for binding to sMF6p/FhHDM-1 in a competitive ELISA in the presence of the entire sMF6p/FhHDM-1 protein (control for maximal inhibition), sFhMF6a, sFhMF6c, or sFhMF6a plus sFhMF6c moieties. We also tested the ability of a large excess of mAb to inhibit these sera. As depicted in Fig 6, the C-terminal region (sFhMF6c, 38.6±3.4% inhibition) induced a higher proportion of antibodies than the N-terminal region (sFhMF6a, 17.0±3.0% inhibition; p<0.01) even though both sequences have a similar length (see Fig 1). Moreover, the inhibition produced by mAb MF6 (30.9±4.7%) indicated that the region corresponding to the epitope recognized by this mAb is antigenic in sheep (Fig 6). Nevertheless, as inhibition by mAb MF6 can produce a phenomenon of steric hindrance over other putative epitopes located in the vicinity (or overlapping the MF6 epitope), its true importance may be lower than indicated by our findings.

### Antibodies produced in sheep to the C-terminal region of MF6p/FhHDM-1 may cross react with *C*. *sinensis*

**Fig 7:**
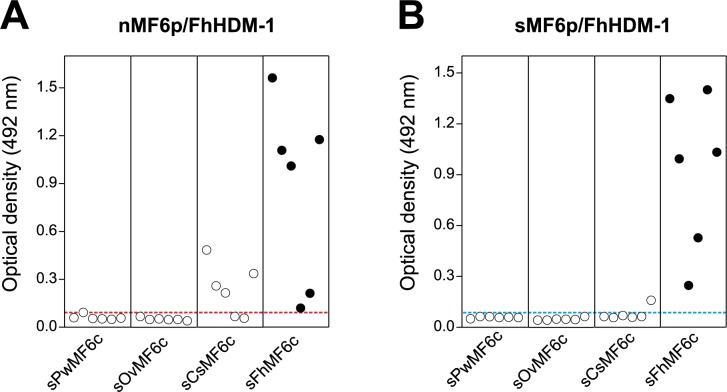
Analysis of the cross-reactivity of the *F*. *hepatica* MF6p/FhHDM-1 protein with orthologs from other trematodes. Serum samples from sheep immunized with either native (A) or synthetic (B) MF6p/FhHDM-1 were tested in indirect ELISA with plates coated with the peptides sFhMF6c (*F*. *hepatica*), sCsMF6c (*C*. *sinensis*), sOvMF6c (*O*. *viverrini*) or sPwMF6c (*P*. *westermani*). Each point in the figure represents the mean OD obtained for one individual serum after subtracting the NSB value (OD = 0.05 ± 0.01). The dotted lined represents a theoretical cut-off value of OD = 0.1. All sera from each category were tested simultaneously by duplicate in the same plate. The intra-assay variation coefficients obtained for each serum did not exceed 7% of the mean OD.

Taking into account the results from mAb MF6 inhibitions performed with peptides derived from the C-terminal region of MF6p/FhHDM-1 orthologs of related trematodes (Fig 3), and the high similarity between these sequences (Fig 1), we tested whether antibodies induced in sheep immunized with either synthetic or native (affinity-purified by mAb MF6) MF6p/FhHDM-1 cross-react with those peptides, in an indirect ELISA. The study was limited to the C-terminal moiety of these proteins since the N-terminal sequences or the entire proteins were not available for the study. As can be observed in Fig 7, some antibodies produced in sheep against the *Fasciola* MF6p/FhHDM-1 protein cross-reacted with the peptide sequence covering the C-terminal moiety of *C*. *sinensis*, but not with the corresponding homologs from *P*. *westermani* or *O*. *viverrini* (Fig 7). Moreover, the data also indicated that the number of cross-reactive sera was higher among those animals immunized with the native protein (Fig 7A) than in those immunized with the synthetic protein (Fig 7B), which suggests that nMF6p/FhHDM-1 shares at least one semiconformational epitope with the protein ortholog from *C*.*sinensis*.

### The antibody immune response to the nMF6p/FhHDM-1 protein in sheep infected by *F*. *hepatica* follows the same pattern as the response induced by L-cathepsins

**Fig 8:**
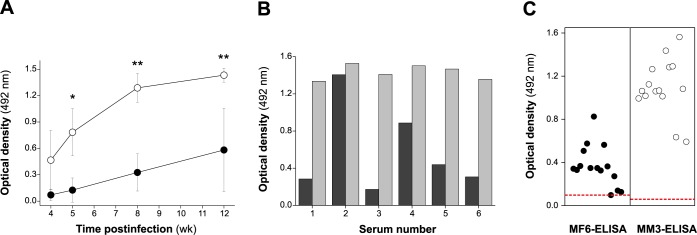
Comparative study of the antibody response obtained in sheep infected with *F*. *hepatica* against nMF6p/FhHDM-1 and L-cathepsins in capture ELISAs. A) Mean antibody response to nMF6p/FhHDM-1 (filled circles) and to L-cathepsins (unfilled circles) of infected sheep (n = 6) along the experimental infection with *F*. *hepatica*. Vertical bars represent the SD of the mean for each value. The Tukey-Kramer multiple comparison test was used to compare the antibody responses of sheep measured by MF6-ELISA and MM3-ELISA. (*): significant differences at p <0.01. (**): significant differences at p <0.001. B) Antibody response against nMF6p/FhHDM-1 and L-cathepsins in individual sheep experimentally infected with *F*. *hepatica* measured at week 12 p.i. C) Individual antibody response of naturally-infected sheep to nMF6p/FhHDM-1 (filled circles) and to L-cathepsins (unfilled circles). Each point in the figure represents the mean OD obtained for each individual serum after subtracting the corresponding NSB value (OD = 0.06 ± 0.01). Horizontal dotted lines show the cut-off values for MF6-ELISA (theoretical OD = 0.1) and MM3-SERO ELISA (OD = 0.074) [11]. The Mann-Whitney test revealed statistical differences between both groups (p <0.0001). Both ELISAs were previously calibrated using serial dilutions of *F*. *hepatica* ESAs and FITC-labeled mAb MF6 or mAb MM3 and HRP-conjugated anti-FITC antibodies as detection system. All sera from each category were tested simultaneously by duplicate in the same plate. The intra-assay variation coefficients obtained for each serum in MF6-ELISA and MM3-ELISA did not exceed 7% of the mean OD.

It has been previously reported that the *F*. *hepatica* MF6p/FhHDM-1 protein is expressed during all phases of the biological cycle of the parasite including ova [23], juvenile forms and adults [22, 23]. In order to investigate the kinetics of the antibody response to this protein, we evaluated the responses produced against the nMF6p/FhHDM-1 by sheep experimentally infected with *F*. *hepatica* in a capture ELISA with mAb MF6. This type of ELISA was preferred to an indirect ELISA with the immunopurified antigens as it should prevent a potential contamination of the sample by L-cathepsins, which are also present in *Fasciola* ESAs. The absence of L-cathepsins in the MF6-capture ELISA was confirmed by the fact that the OD response obtained using biotinylated mAb MM3 (reactive with L-cathepsins), instead of sheep sera, was negligible (not shown). We also tested the response against L-cathepsins produced by *F*. *hepatica* adults in a capture ELISA with mAb MM3 (MM3-SERO). When comparing the responses obtained at weeks 4 to 12 pi (Fig 8A), we observed that the production of antibodies against nMF6p/FhHDM-1 in sheep begins, as for L-cathepsins, around week 4 pi and then increases steadily. However, the response to nMF6p/FhHDM-1 was clearly lower than that of L-cathepsins and varied between different animals (see Fig 8A and 8B). This can also be clearly seen in Fig 8C, in which the antibody responses obtained to nMF6p/FhHDM-1 and L-cathepsins in naturally-infected animals were compared using calibrated capture ELISAs.

## Discussion

Epitope mapping of relevant antigenic proteins from infectious agents is useful for the construction of chimeric vaccines in which sequences from different antigens are grafted together to conform an artificial antigenic molecule. Moreover, mapping of epitopes targeted by mAbs is of interest because the epitopes can be used to investigate whether the mAb-recognized region is involved in the biological activity of the target protein. In the particular case of mAb MF6, in the present study we observed that the sequence of the *F*. *hepatica* MF6p/FhHDM-1 mature protein that is recognized by this mAb covers a small central putative coil region together with some residues located in the amino and carboxy α-helix moieties of the molecule. We have previously observed that, although nMF6p/FhHDM-1 protein present in *F*. *hepatica* ESAs is bound to heme, it can be easily purified by affinity-chromatography with mAb MF6, which strongly suggests that the epitope recognized by mAb MF6 and the heme-binding regions are different. This is consistent with the findings of the present study and those of a recent study [24] in which we observed that, although the heme-binding region of MF6p/FhHDM-1 is also located in the C-terminal region, it specifically comprises the sequence corresponding to the sFhMF6c2 peptide, which only partially overlaps the epitope region of mAb MF6 (sFhMF6c1 peptide) (see Fig 1).

Given the high sequence similarity observed between *Fasciola* and other related trematodes in the region corresponding to the MF6 epitope, we could identify some residues which are probably important for establishing direct bonds with residues in the MF6 paratope. As indicated in the previous section, two of these hot spot residues [35] are ^64^N and ^81^T, as their substitution leads to a decrease or loss of MF6 recognition. We observed that substitution of ^64^N by ^64^G, even maintaining ^81^T, in *C*. *sinensis* and *O*. *viverrini*, was associated to a decrease of more than 50% of the inhibitory activity (see Fig 3A). On the other hand, double substitution of ^64^N by ^64^D and ^81^T by ^81^N led to total loss of recognition, as in *D*. *dendriticum*. Substitution of ^64^N by ^64^D is probably a major change as, although both residues are polar and hydrophilic, ^64^N is neutral and ^64^D is negatively charged. Likewise, the substitution of ^81^T by ^81^N in *D*. *dendriticum* is also consistent with loss of reactivity, as threonine has an available lateral hydroxyl group (see Fig 5B), not available in the asparagine (^81^N) residue (see Fig 5C), which may form a hydrogen bridge with faced residues in the MF6 paratope. The importance of some available key hydroxyl groups in residues involved in the conformation of epitopes recognized by mAbs has already been demonstrated for mAb US9, which recognizes a linear epitope from the gp53 antigen of *Trichinella spiralis* [36].

Binding of the MF6 epitope to the paratope of mAb MF6 probably involves additional residues located on the exposed (hydrophilic) face of the MF6 epitope region (shaded in yellow in Fig 5) that may contribute to the correct spatial positioning of the hot spot residues. In this sense, although in this study we did not perform a structural analysis of the C-terminal peptides assayed, their correct conformation might be acquired by the proper flexibility of the peptides in the aqueous environment or might also be favoured by the binding of mAb MF6 [37].

Comparison of the sequences of the N-terminal and C-terminal moieties of the MF6p/FhHDM-1 protein orthologs present in different related trematodes (i.e., *C*. *sinensis* and *O*. *viverrini* and *D*. *dendriticum*) (Fig 1) revealed a greater number of conserved residues in the C-terminal region. Use of this region as a target for immunodiagnosis may, therefore, cause cross-reactivity. This was confirmed from the experiments performed with sera from sheep immunized with the native or the synthetic MF6p/FhHDM-1 proteins, which showed that: i) the proportion of antibodies reactive to the C-terminal region of the molecule was higher than the numbers directed at the N-terminal region (Fig 6), and ii) antibodies induced in some animals to the nMF6p/FhHDM-1 protein cross-reacted with the peptide corresponding to the C-terminal region of the MF6p/FhHDM-1 ortholog from *C*. *sinensis* (Fig 7). The antigenicity of the MF6p/FhHDM-1 protein orthologs in different trematode infections has not been investigated in detail. However, in the case of *C*. *sinensis* it has been reported that between 71.9 and 81.3% of serum samples from patients infected with this parasite were able to recognize this protein in ELISA and immunoblotting, respectively [38]. The same authors also indicated that between 35.3% (ELISA) and 47.1% (immunoblots) of serum samples from patients with *P*. *westermani* infections cross-reacted with the MF6p/FhHDM-1 protein ortholog from *C*. *sinensis* but, contrary to what we observed in our study with immunized sheep, no cross-reactivity was observed with sera from patients infected with *F*. *hepatica*. One possible explanation for this discrepancy with respect to human studies is that natural human infections by *F*. *hepatica* may produce different amounts, or a different set of antibodies, than sheep immunized with the *Fasciola* MF6p/FhHDM-1 protein.

As indicated above, the cross-reactivity among MF6p/FhHDM-1 protein orthologs from different trematodes and their relatively low sensitivity as targets in ELISA or immunoblotting [38] probably limit their use in serodiagnostic tests. However, as MF6p/FhHDM-1 is produced throughout the entire life cycle of *F*. *hepatica* [22, 23], we investigated the antigenicity of this protein in infected sheep, as well as whether the antibody response to such protein precedes the response induced by L1, L2 and L5 cathepsins, which are mainly produced by immature and adult forms of the parasite [15, 16, 20]. As indicated in the previous section, the antibody levels in either experimental or natural *Fasciola* infections were much lower for MF6p/FhHDM-1 protein than for L-cathepsins and, as deduced from experimental infections, the antibody response to the MF6p/FhHDM-1 protein did not precede that obtained against L-cathepsins. These data are consistent with those of a preliminary study carried out by Robinson et al. [22] in which serum samples from sheep infected with *F*. *hepatica* were tested and further analyzed by ELISA and Western blot. Together, these results confirm that the MF6p/FhHDM-1 protein is not a good antigen for serodiagnosis of *Fasciola* infections. However, this does not limit their possible usefulness in the elaboration of multicomponent and/or chimeric vaccines against *Fasciola* and related trematodes. Inclusion of this protein in the formulation of novel vaccines to prevent *Fasciola* infections may be of interest for at least two reasons: i) MF6p/FhHDM-1 has already been shown to induce partial protection in cattle [39]; and ii) this protein has been implicated in heme homeostasis in the parasite [23, 24] and has been shown immune suppression capabilities, which may favor parasite survival [25, 40, 41]. Therefore, neutralization of the protein by antibodies and/or other immunological mechanisms may be deleterious for the parasite. Although in this study we observed that the synthetic protein was not well recognized by sera from sheep naturally or experimentally infected with *Fasciola* (not shown), we obtained a good recognition with sera from animals immunized with a formulation containing either native or synthetic MF6p/FhHDM-1 and an adjuvant (Quil-A). This result suggests that the synthetic protein is partially misfolded but that the presence of the adjuvant forces the immune system to target additional epitopes not usually recognized in the native protein during natural infections due to the immunodominance of discontinuous conformational epitopes. This finding is of interest for the development of anti-*Fasciola* vaccines that include *Fasciola* MF6p/HDM proteins, as it shows that the native and the synthetic proteins are immunogenic and may even produce a complementary set of antibodies.

In summary, we demonstrated that mAb MF6 recognizes a continuous conformational (semiconformational) epitope located within the C-terminal moiety of the *Fasciola* MF6p/FhHDM-1 and MF6p/FgHDM-1 proteins, and that this region is more antigenic in sheep than the corresponding N-terminal moiety. Considering that antibodies directed to the C-terminal moiety of these *Fasciola* proteins cross-reacted with MF6p/HDMs from other trematodes (e.g., *C*. *sinensis*), and that these proteins are poorly recognized during natural infections, we conclude that *Fasciola* MF6p/HDM proteins are not good antigen candidates for serodiagnosis of *Fasciola* infections. Nevertheless, taking into account the physiological relevance of these proteins in heme homeostasis, and their reported immunomodulatory properties, either the entire protein, or the C-terminal region containing the MF6 epitope and the heme-binding region, may be of potential interest as targets for development of new chimeric or multicomponent vaccines. In this regard, preliminary studies in our laboratory showed that immunization with sMF6p/FhHDM-1 prior to infection of sheep decreases the production of L-cathepsins by adult flukes.
